# Ownership and utilization of insecticide-treated nets (ITNs) for malaria control in Harari National Regional State, Eastern Ethiopia

**DOI:** 10.11604/pamj.2015.21.52.5380

**Published:** 2015-05-25

**Authors:** Zelalem Teklemariam, Aymere Awoke, Yadeta Dessie, Fitsum Weldegebreal

**Affiliations:** 1 Haramaya University, College of Health and Medical Sciences, Department of Medical Laboratory Sciences, Harar, Ethiopia; 2 Haramaya University, College of Health and Medical Sciences, Department of Environmental Health, Harar, Ethiopia; 3 Haramaya University, College of Health and Medical Sciences, Department of Public Health, Harar, Ethiopia

**Keywords:** Insecticide-treated bed nets (ITNs), Harari, malaria, utilization, ownership

## Abstract

**Introduction:**

Insecticide-treated nets (ITNs) stood at center in the current efforts to prevent and control malaria at community and individual levels. Though ITNs are the most prominent measure for large-scale deployment in highly endemic areas their compliance in terms of ownership and usage needs attention. The aim of this study was therefore to determine the ownership and utilization pattern of ITNs in Harari Peoples National Regional state, Ethiopia.

**Methods:**

A community based cross-sectional study was conducted in Harari National Regional State from September to October, 2012. A total of 784 households were included from malarious areas. Data were collected by using structured questionnaires and observational checklist.

**Results:**

About 57.9% of participants had at least one ITNs. The utilization of ITNs based on history of sleeping under net in the previous night was 73.3%. Regarding proper use of ITNs, 57.9% of respondents demonstrated proper hanging and tucking. Those households with secondary school education (AOR: 1.775(1.047, 3.009)), knowledge about ITNs use (AOR: 2.400(1.593, 3.615)) and knowledge of malaria transmission by bite of mosquito (AOR: 1.653(1.156, 2.365)) have more likely hood to own ITNs.

**Conclusion:**

ITNs Ownership was low as compared to the target by Federal ministry of Health of Ethiopia. Though utilization of ITNs was promising, there are still significant number of participants who demonstrate hanging and tucking improperly. Therefore, health bureau need to work towards increasing the distribution of ITNs per household and also provide health information through health extension workers to enhance regular and proper usage of the ITNs.

## Introduction

Malaria is one of the major public health problems particularly in sub-Saharan Africa where an estimated 90% of all cases [1] and 20.3% of all deaths of under five children [2] occur. It contributes to increased poverty, which causes for annual losses of up to 12 billion dollars in Sub-Saharan African countries [3]. The World Health Organization (WHO) estimated that the number of cases of malaria rose from 233 million in 2000 to 244 million in 2005 but decreased to 225million in 2009. The number of deaths due to malaria is estimated to have decreased from 985,000 in 2000 to 660,000 in 2010 [4].

Ethiopia is one of those severely affected countries by malaria. Three-forth (75%) of the land in the country is malarious and 68% of the population is at risk of malaria infection [5, 6]. Malaria was the first cause of outpatient visits and the second cause of health facility admissions with the rate of 12% and 9.9% respectively according to 2007/2008 Federal Ministry of Health report [7]. It has a significant problem to social and economic development of the country due to its epidemicity occurring during harvesting seasons. This reduces agricultural productivity, hence leads to food insecurity and poverty [5]. Insecticide Treated Nets (ITNs) for personal protection of vulnerable groups and community protection has been employed as main vector control tools in Ethiopia. Since 1997, ITNs were distributed to different parts of the country to control the transmission of malaria [5, 6, 8]. The use of ITNs is a cost-effective intervention to reduce child mortality and maternal anemia where malaria imposes an important disease burden [9].

ITNs are being distributed in all malarious areas of Ethiopia with the assistance of health extension workers, volunteer community workers and local administration. However, even if all households at risk are fully covered, nets must also be used consistently and correctly if they are to have maximum impact [10]. The coverage and proper utilization ITNs which is one of the most promising malaria preventive measure in the country is also limited due to lack of sustainable distribution and issues related to replacement of nets, seasonality of malaria, and poor knowledge of the community with regard to the link between mosquitoes and malaria [8]. The coverage and utilization of ITNs were varying from place to place in Ethiopia [10–17]. But there is no published data on Harari National Regional State. Therefore, this study determined the ownership and utilization of ITNs in Harari National Regional State, Eastern Ethiopia.

## Methods

### Study area, design and period

Harari National Regional State, which is one of the regional states of Federal Democratic Republic of Ethiopia, is located at East part of Ethiopia at distance of 515km from Addis Ababa. It is neighbored to the north with Kombolcha and Jarso, to the east with Gursum and Babile Woreda, in the south with Fedis and in the west with Haramaya Woreda of the Oromia regional state. The health service coverage is estimated to be about 100%. There are 6 Hospitals (2 military, 1 private, 1 Non-Governmental and 2 governmental) and 8 health centers in the region. The region has 8 worades and 36 kebeles (17 rural and 19 urban) [18]. A community based cross-sectional study was conducted to determine the ownership and utilization of Insecticide treated nets in Harari National Regional State from September - October 20/ 2012.

### Sample size and sampling techniques

The sample size was determined by using single proportion formula by setting 95% confidence interval, P= 0.5, margin of error 0.05 and design effect of 2. Then, 5% non-response rate was added. The final sample size was 806. To select 806 households multistage random sampling technique was employed. It was initially distributed proportionally based on population size to each of the 8 woradas in the region. About 55(7%), 241 (30.7%), 144(18.4%), 106(13.5%), 96(12.2%), 43(5.5%), 33(4.2%) and 66(8.4%) of the sample size was taken from Erer, Sofi, Deri-Teyara, Amir Nur, Shenkor, Aboker, Jenila and Hakim woreda, respectively. Then, sample size allotted for each woreda was distributed proportionally based on population size to malarious kebeles under it. In the final stage households were selected from each kebeles by systematic random sampling techniques from random starting point until the allotted sample size fulfill.

### Data collection

Data were collected by trained final year nursing students of Haramaya University by using structured questionnaires and observational checklist. The questionnaire was designed to collect information on socio demographic characteristics, net ownership, family size, knowledge about mosquito nets, perception about the use of nets and others. It was adopted from the questionnaire used in another study in Ethiopia [13]. First, it was prepared in English and then translated to local languages. Pre-test was conducted outside the study area which used check the validity of our questionnaire, reaction of the respondents, language barriers, time needed for administering the questionnaire and to make amendments if needed. House to house interviews were conducted with the participating household heads or another adult in the house (if the household head was absent or unable to respond for any reasons). Mosquito net tucking was assessed for those household who had ITNs.

### Data analysis

Data were entered into Epidata Version 3.1 after checking their completeness by the research teams. Then, it was transported to SPSS 16.0 statistical package for windows for analysis. Descriptive statistics were used to examine the study variables. Univariate and bivariate analyses were performed to determine factors affecting ITN ownership and utilization by households as an outcome variable. Crude and adjusted odds ratio and their 95% confidence interval were used to examine the strength of association. A statistical test result was reported as significant when its p value was less than 0.05

### Ethical consideration

Ethical clearance was obtained from Haramaya University Colleges of Health and Medical Sciences Institutional Health Research and Ethical Review Committee. Prior to data collection regional health bureau, local officials and the community were approached through formal letters written from the Haramaya University. All the participants were explained about the purpose and their right to participate or not to participate in this study. Those who gave their written consent were participated in this study. Moreover, all personal information of the participants was kept confidential.

## Results

**Socio-demographic characteristics of the study participants:** a total of 784 households were participated in the study and the response rate was 97.3%. The average family size was 4.5(SD +2) and the range was 1-19. The mean age of the household participant was 37.6(SD +17.0) and the range was 16 - 99. Most of the participant was female (66.8%), farmer(33%) and elementary school (36.6%) (Table 1).

**Table 1:** Characteristics of study participant households in Harari National Regional State, eastern Ethiopia, 2012

Socio demographic characteristics	No (%)
**Sex**	
Male	260(33.2)
Female	524(66.8)
**Occupation**	
Government employee	82(10.5)
Merchant	100(12.8)
Farmer	259(33)
Student	99(12.6)
Private employee	35(4.5)
Daily laborer	39(5)
Other	34(4.3)
No job	117(14.9)
**Educational status**	
Cannot read and write	197(25.1)
Read and write	79(10.1)
Elementary school(1-8)	282(36.6)
Secondary school 9-12	195(25.3)
Tertiary (college/university)	18(2.3.)

**Knowledge and perception of households about of insecticide treated nets (ITNs):** about 17.5% of the household respond as one of their family member had been infected with malaria during past one year. About 61.1%, 39.0%, 63.4% and 8.7% of the households responded for the use of ITNs as it repel (avoidance of biting of mosquitoes), kill mosquitoes, prevent malaria and kill fleas/ bugs respectively. Those households were categorized as they have knowledge about use of ITNs, if he/she respond at least one use of ITNs correctly from repel (avoidance of biting of mosquitoes), kill mosquitoes, and/or prevent malaria. Based on that the overall knowledge of household about ITNs use was 84.2%. Most (83.1%) of the participants responded as there is low risk of getting malaria while sleeping under ITNs. About 76.6% of the households responded to know as malaria is transmitted by bite of mosquitoes (Table 2). When asked if you have one ITNs who will be given priority to sleep, 19.0%,12.1% and 37.5% of them replied for wife and under 5 child, wife and husband and as they don't know respectively. The least priority given group was pregnant women (1%) (Table 3).

**Table 2:** Knowledge and perception of households about ITNs and malaria transmission in Harari National Regional State, eastern Ethiopia, 2012

Characteristics	n = 784 no(%)
**At least one family member infected with malaria in past one year**	
Yes	137(17.5%)
No	647(82.5)
**Knowledge of respondent about use ITN**	
Repeal mosquito (avoidance of biting of mosquitoes)	479(61.1)
Kill mosquito	306 (39)
Prevent malaria	497(63.4)
Kill fleas/bugs	68(8.7)
Protect person from dust/dirt	8(1.0)
I don't know	14(1.8)
Other	1(0.1)
**Can someone get malaria while sleeping under ITNs**	
Yes	126(16.9)
No	614 (83.1)
**Knowledge about malaria transmission by mosquito bite**	
Yes	599(76.6)
No	185(23.6)

**Table 3:** Responses of household “who get priority if there is only one ITNs to sleep under it?” in Harari National Regional State, eastern Ethiopia, 2012

If you have only one ITNs who can get priority to sleep under it	Number (%)
wife and child under 5, husband, wife and child under 5	213(44.3)
husband and wife	95(19.3)
younger children(b/n5-14)	72(14.7)
Wife	48(9.8)
youths(age > 15)	26(5.3)
Husband	18(3.7)
Other	10(2.0)
elderly/grand parents	8(1.6)
pregnant women	1(0.2)

**Figure 1:**
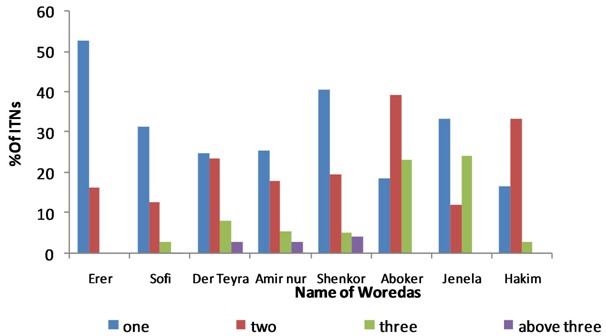
Number of ITNs among respondents household by woreda in Harari National Regional State, eastern Ethiopia, 2012

**Ownership and utilization of ITNs:** about 57.9% of household participants had at least one ITNs. Most of the other households had responded as they had ITNs sometime in the past but they have lost it for unknown reason. Further stratified analysis indicated that the percentage of households who owned 1, 2 and 3 nets were 30.2%, 19.8% and 6.4%, respectively. The average number of ITNs per household was 1.4 (range 0-6). Most (51.8%) of the study participant had gotten ITNS before 6 months (Table 4). The highest ownership with one ITNs were reported from households in Erer and Shenkor Worada (Figure 1). About 99% (402/406) obtain ITNs free of charge. The average utilization of ITNs based on history of sleeping under net in the previous night was 73.3%. Several reasons were ascribed for not sleeping under the net in the previous night, of which 69.9% and 1.9% of the household respond as there is no mosquito in their area and they didn't know how to use ITNs, respectively. Regarding proper use of ITNs, 57.9% of respondents from those who have the ITNs demonstrated proper hanging and tucking (Table 4). The frequency of usage of ITNs responded by household to be daily, summer (May-August), spring (September-November), winter (December-February), occasional and weekly was 249/454(54.8%), 41/454 (9.0%), 6/454 (1.3%), 50/454 (11.0%), 91/454 (20.0%), and 2/454 (0.4%) respectively.

**Table 4:** Ownership, utilization, reasons for not using, demonstration of proper use of ITNs by household in Harari National Regional state, Eastern Ethiopia, 2012

Characteristics	No (%)
**Number of ITNs per household**	
One	237(30.2)
Two	155(19.8)
three	50(6.4)
Greater than three	12(1.5)
**Had you slept under ITNs last night**	
Yes	333(73.3)
No	121(26.7)
**Reasons for not sleeping under ITNs last night**	
It is too hot to use	5(4.9)
There are no mosquito	72(69.9)
Nets don't suit their house	9(8.7)
It is not the season	5(4.9)
Don't know how to use	2(1.9)
It is dirty	5(4.9)
Other	5(4.9)
**Demonstration of proper use ITNs (n = 454)**	263(57.9)
**When had you get the ITNs?**	
Before 6 months	218(51.8)
Before 6 -12 months	73(17.3)
Before 1-2 years	83(19.7)
Before More than 2years	35(8.3)
I don't know	12(2.9)

**Determinants of ITNS Ownership and utilization by households:** those households who are students have more ITNs than other occupational status (COR: 0.447(0.205,0.971)). Households with Secondary school educational level have more ITNs than other educational status (COR: 2.236(1.399, 3.573)). Those who have Knowledge about ITNs use (COR: 1.556(1.141, 2.122)) and knowledge of malaria transmission by bite of mosquito ((COR: 1.782(1.278, 2.484)) own more ITNS. In addition, those household having at least one family member infected with malaria during past one year have more ITNs (COR: 1.501(1.037, 2.172)). In the final adjustment, only those households with secondary school education (AOR: 1.775(1.047, 3.009)), knowledge about IINs use (AOR: 2.400(1.593, 3.615)) and knowledge of malaria transmission by bite of mosquito (AOR: 1.653(1.156, 2.365)) are more likely to own ITNs (Table 5). Households who are Farmers in occupation (COR: 3.061(1.525, 6.145)) and Secondary school in educational status (COR: 2.236(1.399, 3.573)) were less utilize ITNs. Those household, who have knowledge about ITNs use were less likely to utilize ITNs (COR: (0.352(0.146, 0.849)). In the final adjustment those farmer were more likely to utilize ITNs than other occupation (AOR: 2.262(1.002, 5.108)). Those household who have knowledge about ITNs use were less likely to utilize ITNs (AOR: 0.373(0.151, 0.918)) (Table 6).

**Table 5:** Regression analysis for ownership ITNs by households in Harari National Regional state, Eastern Ethiopia, 2012

Variables	Ownership of ITNS		Crude odds ratio (95% CI)	P value	Adjusted odd ratio (95% CI)	P value
	Yes n (%)	No n (%)				
**Occupation**						
Government employee	57(69.5)	25(30.5)	1		1	
Merchant	60(60.0)	40(40.0)	0.950(0.396,2.278)	0.909	0.758(0.384,1.493)	0.423
Farmer	134(51.7)	125(48.3)	0.625(0.270,1.447)	0.272	0.674(0.361,1.258)	0.215
Student	63(63.6)	36(36.4)	0.447(0.205,0.971)	0.042	0.845(0.441,1.620)	0.611
Private employee	21(58.3)	15(41.7)	0.729(0.314,1.695)	0.463	0.638(0.269,1.513)	0.308
Daily laborers	21(53.8)	18(46.2)	0.583(0.216,1.572)	0.287	0.512(0.219,1.200)	0.123
Has no job	62(53.0)	55(47.0)	0.486(0.184,1.282)	0.145	0.625(0.323,1.207)	0.162
Other	24(70.6)	10(29.4)	0.470(0.206,1.069)	0.072	1.256(0.488,3.234)	0.637
**Educational status**						
Cannot read and write	104(52.8)	93(47.2)		1		
Read and write	40(50.6)	39(49.4)	0.917(0.544, 1.546)	0.746	0.868(0.503,1.496)	0.610
Elementary school(1-8)	161(57.1)	121(42.9)	1.190(0.825, 1.715)	0.352	1.131(0.763,1.676)	0.541
Secondary school (9-12)	95(71.4)	38(28.6)	2.236(1.399, 3.573	0.001	1.775(1.047,3.009)	0.033
Tertiary(college/university)	49(61.2)	31(38.8)	1.413(0.832, 2.401)	0.200	1.005(0.534,1.893)	0.987
**Knowledge about the use of ITNs**						
Yes	403(61.1)	257(38.9)	1.556(1.141,2.122)	0.005	2.400(1.593,3.615)	0.000
No	73(58.9)	51(41.1)	1.000		1	
**Knowledge about malaria transmission**						
Yes	367(61.3)	232(38.7)	1.782(1.278,2.484)	0.001	1.653(1.156,2.365)	0.006
No	87(47.0)	98(53.0)	1.000		1	
**At least one family member caught malaria in past one year**						
Yes	68(49.6)	69(50.4)	1	0.031	1	0.116
No	386(59.7)	261(40.3)	1.501(1.037,2.172)		1.379(0.924,2.059)	

**Table 6:** Regression analysis for utilization ITNs by households in Harari National Regional state, Eastern Ethiopia, 2012

Variables	Utilization of ITNS		Crude odds ratio (95% CI)	P value	Adjusted odd ratio (95% CI)	P value
	Yes n (%)	No n (%)				
**Occupation**						
Government employee	35(61.4)	22(38.6)	1		1	
Merchant	39(68.4)	18(31.6)	1.362(0.629, 2.948)	0.433	1.171(0.507,2.705)	0.711
Farmer	112(83.0)	23(17.0)	3.061(1.525, 6.145)	0.002	2.262(1.002,5.108)	0.049
Student	43(69.4)	19(30.6)	1.423 (0.666, 3.038)	0.363	1.287(0.579,2.857)	0.536
Private employee	17(70.8)	7(29.2)	1.527(0.545, 4.272)	0.420	1.224(0.418,3.580)	0.712
Daily laborer	17(85.0)	3(15.0)	3.562(0.934, 13.579)	0.063	3.049(0.759,12.249)	0.116
Has no job	41(67.2)	20(32.8)	1.289(0.606, 2.742)	0.510	1.024(0.447,2.346)	0.955
Other	17(68.0)	8(32.0)	1.336(0.494, 3.614)	0.569	1.108(0.375,3.274)	0.853
**Educational status**						
Cannot read and write	80(79.2)	21(20.8)	1		1	
Read and write	31(70.5)	13(29.5)	0.626(0.279, 1.402)	0.255	0.770(0.329,1.804)	0.548
Elementary school(1-8)	125(75.8)	40(24.2)	0.820(0.451, 1.492)	0.516	0.926(0.485,1.769)	0.816
Secondary school 9-12	68(73.9)	24(26.1)	0.744(0.381, 1.452)	0.386	1.006(0.470,2.155)	0.988
Tertiary(college/university)	25(52.1)	23(47.9)	0.285(0.136, .600)	0.001	0.430(0.180,1.025)	0.057
**Knowledge about ITNs use**						
No	43(87.8)	6(12.2)	1	0.020	1	0.032
Yes	290(71.6)	118(28.4)	0.352(0.146,0.849)		0.373(0.151,0.918)	

## Discussion

In this study about 57.9% of the households have at least one ITNs. This is similar to report of 56.6% in South west Ethiopia [16] and World Malaria Report of 2011 (56%) [19]. It was higher than report from studies conducted in Ethiopia: Oromia and Southern Nation Nationalities people Region (SNNPR) (47.5%) [11] and other areas (37%) [12]. It was slightly lower than the target set to cover 60% of households in 2007 by the Federal Democratic Republic of Ethiopia Ministry of Health (FMOH) [6] and another study from Gursum, Eastern Ethiopia (62.4%) [10]. This result was also lower than other studies conducted in selected malaria prone area of Ethiopia (81.3% to 99.3%) [13], national malaria indicator survey of 2007 (65.6%) [14], Kafta - Humera- Tigray (85.5%) [15], Afar (86.6%) [17] and in other African countries e.g. Kenya (75%) [20] and Sierra Leone (83.4%) [21]. It is also lower than ITNs coverage recommended by WHO for an acceptable level of protection [19]. The mean number ITNs per household were 1.4 ITNs per household. It is lower than 1.7 per household report in South west Ethiopia [16] and national plan of 2 per household at the end of 2007 [22].

There is great variation on ITNs coverage when this study compared to other studies. However, those households who have not ITNs responded that they had sometime in the past but lost it for unknown reason. Even most of those respondents who had ITNs reported as they got it before 6 months of this study. This implies that the importance of monitoring of ITNs ownership in each household by health extension workers working in the community is vital. The issues of replacing those worn or lost ones and making available for those who had not any should also be considered by the regional health bureau for effective prevention and control of malaria. It is known that ITNs efficacy depends on regular and proper utilization [10]. In this study 73.3% of those households with ITNs reported as they were slept under ITNs in the previous night. This was higher than report from other studies in Ethiopia: Oromia and SNNPR (35.4%) [11], Gursum (21.5%) [10] and national malaria indicator survey of 2007(53.2%) [14]. It is also higher than study conducted in Kenya (46.7%) [20] and Sierra Leone (67.2%) [21]. But it was lower than 80% utilization recommended by WHO [19]. Various reasons were ascribed by households for not utilizing the ITNs. Some of them include the perceptions of the absence of mosquitoes in their area, too hot to sleep under the net, dirtiness of nets, improper fittings of nets with the available sleeping space and others. Similar findings and explanations were provided for non-utilization of the available ITNs in the studies conducted in Kenya and SNNPR, Ethiopia [20, 23].

The majority of the respondents, who had ITNs, noticed benefits obtained from using ITNs. The reported benefits include protection from malaria, repelling off or killing of mosquitoes. In the five year strategic plan for malaria prevention and control in Ethiopia, it was indicated that ITNs are useful to control malaria through either prevention of mosquito bites or prevention of disease [22]. Similar finding from a study conducted in western Kenya reported that most respondents liked ITNs because they protect against malaria and some others stated they liked bed nets because they kept off and killed mosquitoes [24]. However some respondents reported the benefit of ITNs as to kill fleas/bugs, protect from dust and they don't know at all. This might have an impact on the diversion of net for other purpose or initiate a person not to use the net. ITNs ownership and utilization can be affected by different characteristics. In this study those households with secondary school educational status are more likely to own more ITNs than others. This might be due to those respondents might get information about ITNs in their secondary school educational curriculum. Most of the households have knowledge about ITNs use and malaria can be transmitted by mosquitoes bite. This slightly lower than study conducted in Afar [17] and SNNPR, Amhara and Oromiya [25] which showed about 89.5% and 93% of respondents knew that malaria could be transmitted through mosquito bite, respectively. Those households, who have knowledge about ITNs use and knowledge of malaria transmission by bite of mosquito, are more likely to own more ITNs than others. This is similar to other study in Ethiopia [10].

Those farmers were more likely to utilize ITNs than respondents with other occupational status. This is different from other study in Ethiopia [10]. It appears those who have knowledge about ITNs use are less likely to utilize ITNs. This might be due to the households might not be able to properly hang and tuck nets or not used ITNs at the time of study only. From the households that own ITNs, only 57.9% of those household with ITNs demonstrated proper hanging and tucking of nets in this study. This is higher than 21.5% report from Gursum, eastern Ethiopia [10]. However, the proportion of respondents who demonstrated proper use of ITNs was less than those who used their nets the previous night (73.3%). Most of the households were also using their ITNs daily (54.8%), while some of them were using occasionally (20%). This is more or less similar to the previous study conducted in Ethiopia [26]. But, it was higher than the base line study made in Ethiopia for frequency of ITNs usage [25]. Therefore, this gap should be filled in order to improve the proper utilization of ITNs in the region.

## Conclusion

In general, in this study the ownership of ITNs was slightly lower than a target set by FMOH. Households with Secondary school educational status, knowledge about ITNs use and the transmission of malaria by mosquitoes bite are factors that determine the ownership of ITNs by households in the region. The utilization of ITNs found promising in this study. Those farmers were more likely to utilize ITNs. Those household, who were able to properly use ITNs and use them daily, were slightly more than 50%. Therefore this study recommends, Health bureau should work to increase the distribution of ITNs per household by using health extension worker. The health extension workers should also demonstrate each household how to properly hang on their house at the time of distribution of ITNs. They must also work on the sustainability of the use of ITNs and other malaria prevention and control measures via increased community participation. In addition, they should provide continues health information and monitoring of the ITNs usage by household in order to enhance regular and proper usage of net. Finally, this study also recommends further study about the current magnitude of malaria and mosquito infestation in the region.
